# Seroprevalence of canine distemper virus (CDV) in the free-roaming dog *(Canis familiaris)* population surrounding Chitwan National Park, Nepal

**DOI:** 10.1371/journal.pone.0281542

**Published:** 2023-02-27

**Authors:** Inga McDermott, Martin Gilbert, Manoj Kumar Shah, Amir Sadaula, Neil E. Anderson

**Affiliations:** 1 The Royal (Dick) School of Veterinary Studies and the Roslin Institute, University of Edinburgh, Roslin, United Kingdom; 2 Cornell Wildlife Health Center, College of Veterinary Medicine, Cornell University, Ithaca, NY, United States of America; 3 Faculty of Animal Science, Veterinary Science and Fisheries, Agriculture and Forestry University, Rampur, Chitwan, Nepal; 4 National Trust for Nature Conservation (NTNC), Vinayak, Godawari, Nepal; University of Reunion Island, RÉUNION

## Abstract

Canine distemper virus (CDV) is a global multi-host pathogen that is capable of causing considerable mortality in a range of species and is important in the field of conservation medicine. Nepal’s Chitwan National Park is a protected area providing habitat for 32% of the country’s mammal species including endangered carnivores such as the Bengal tiger *(Panthera tigris tigris)* that are susceptible to CDV. The presence of free-roaming dogs around protected areas could represent a source of infectious disease for transmission to local wildlife. A cross-sectional demographic and canine distemper virus seroprevalence study of 100 free-roaming dogs from the Chitwan National Park buffer zone and surrounding area was conducted in November 2019. The overall seroprevalence indicating past exposure to canine distemper virus was 80.0% (95% CI: 70.8–87.3). Of the host variables assessed, sex and age were positively associated with seroprevalence at the univariable level, with male dogs demonstrating lower seroprevalence than females (OR = 0.32, 95% CI: 0.11–0.91) and adult dogs demonstrating higher seroprevalence than juveniles (OR = 13.94, 95% CI: 1.37–142.29). The effect of sex was no longer significant at the multivariable level, but the direction of the effect remained the same. The effect of age remained significant after multivariable analysis (OR = 9.00, 95% CI: 1.03–192.75). No spatial associations were demonstrated in relation to the buffer zone area or boundary of Chitwan National Park. Free-roaming dog neutering and vaccination programmes can provide a useful baseline for future CDV studies in the region, and a proxy to monitor disease threats to susceptible wildlife.

## Introduction

Canine distemper virus (CDV) was initially regarded as a pathogen only affecting the domestic dog but is now recognised as a globally distributed multi-host pathogen that causes high mortality in a wide range of carnivore species, making it an important disease in the field of conservation medicine [1, 2].

Canine distemper virus, a negative sense, single-stranded RNA virus within the family *Paramyxoviridae*, is classified within the genus *Morbillivirus* which contains other highly pathogenic viruses including measles virus affecting humans and other primates, *peste des petits ruminants* virus affecting small ruminants, phocine and porpoise distemper viruses affecting marine mammals, and rinderpest virus which affected artiodactyls prior to its eradication in 2011 [2–6]. With an almost worldwide distribution [7], CDV shares the characteristics of other RNA viruses. Low copy fidelity during replication produces mutations, resulting in infection and transmission by a wide range of hosts and contributes to CDV’s status as an important emerging pathogen [6, 8].

Following inhalation of infectious aerosol droplets, the virus multiplies in alveolar macrophages. Once viral replication is initiated, spread occurs to all lymphoid tissue where continued multiplication causes severe lymphodepletion and immunosuppression. Epithelial colonisation of virus, commonly in the respiratory and gastrointestinal tracts, results in systemic disease and clinical signs producing viral shedding in both clinical and sub-clinical infections [2, 9]. Viral shedding occurs mainly via the oronasal route by aerosol and is highly contagious. The virus degrades rapidly in the environment (although viability may be extended at lower temperatures), and so transmission is largely dependent on direct close contact between infected and susceptible hosts [2, 3].

A strong, humoral, and cellular immune response during the early stages of infection can eliminate virus from all tissues ceasing viral shedding and some hosts can recover completely [9]. In individuals that are unable to mount an adequate immune response, CDV can spread to invade the central nervous system and can result in death from encephalitis [3]. Recovered hosts cease viral shedding and potentially have life-long immunity to subsequent CDV infections [3, 10, 11]. Protective maternal antibodies are passed vertically to the young, the levels of which reduce over time and are completely lost by 16 weeks of age [11–13]. There is only one recognized serotype although there are several genotypes which relate to geographical regions and genetic drift alterations of the external Haemagglutinin glycoprotein [1, 2, 7]. Preventative vaccination against CDV in domestic dogs has seen success since the 1960’s leading to the control of the disease in some countries, but sufficient vaccine coverage is required to avoid re-emergence of disease [10, 14].

Most dogs with owners in Nepal are allowed to roam freely, and what entails ownership can vary widely [15–18]. The size of the free-roaming dog population is supported by rapid city growth and urbanisation, poor or absent garbage policy, open slaughter facilities and reduced scavenger competition following the 1990’s decline of vulture populations [15, 19–22]. Free-roaming dogs can have negative effects on wildlife through their interactions as prey, competitors, disease reservoirs and vectors, and frequently as predators [18, 23–25]. Country-wide dog census information is lacking in Nepal [15, 17, 26, 27], but in 1998 the National Zoonoses and Food Hygiene Research Centre (NZFHRC) estimated two million dogs were present in Nepal, which has a human population estimated at 28 million [15, 16].

With a short infection cycle causing high mortality or life-long immunity, CDV infection in small stable populations (such as those of many endangered species) should fade-out over time as the effective reproductive number falls below one (R_T_ <1) [28]. However, for multi-host pathogens like CDV, transmission from more abundant reservoir hosts with high population turnover can act as a continued source of infection potentially reducing endangered populations to the point of extinction [1, 2, 8, 28–32]. One of the most famous wildlife outbreaks occurred in 1994, when an outbreak of CDV in the lion population in Serengeti National Park coincided with the loss of an estimated 1000 animals [33–35]. CDV has now also been identified in African wild dogs [36], and wild tigers in Russia and India [37, 38].

Chitwan District supports a large free-roaming dog population which, if engaging in direct or indirect contact with wildlife, could pose a risk of pathogen transmission at disease interfaces. Chitwan National Park (CNP) provides habitat to endangered carnivores including the Bengal tiger *(Panthera tigris tigris*) and Asiatic wild-dog (*Cuon alpinus*), for which exposure to CDV could pose a conservation threat. Canine distemper virus is common in free-roaming dogs in other countries [39–43], and is considered endemic in Nepal but has been little studied in domestic or wildlife species [44], with only one published study providing CDV seroprevalence data from domestic dogs in the Annapurna region [45], and one serosurvey of eleven Bengal tigers from CNP [46].

This study generated demographic and baseline seroprevalence data for CDV in the free-roaming dog population surrounding CNP, Nepal. Variables which might be important in understanding the epidemiology of the virus were investigated for association with CDV seroprevalence. The distance from dogs’ resident locations to the CNP boundary were also investigated to establish if proximity to urban human habitations was associated with higher exposure to CDV. Our findings provide a foundation for further CDV research in this important conservation area.

## Materials and methods

### Study area

**Fig 1:**
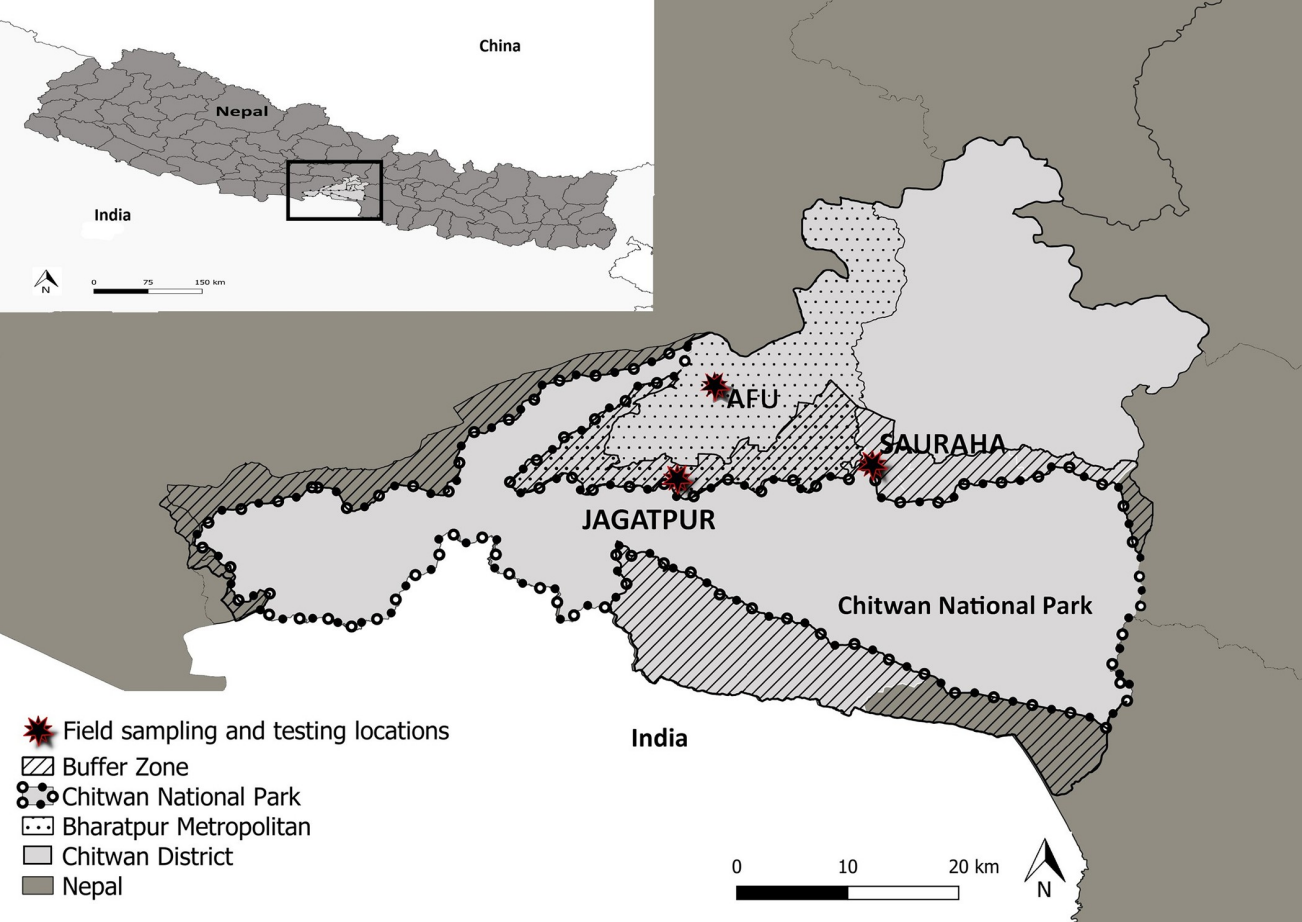
Map of Nepal and Chitwan District study location. The main map shows the study locations within Chitwan District, with Chitwan National Park, the buffer zone and Bharatpur Metropolitan City highlighted. The inset map illustrates the location of Chitwan District within Nepal.

Chitwan National Park is situated in the central subtropical inner Terai lowlands (27.5341° N, 84.4525° E), and was designated as a protected area (PA) in 1973 and a UNESCO World Heritage site in 1984 (Fig 1). Covering 932 km^2^, CNP provides habitat for 68 (32%) of Nepal’s mammal species, of which 22 (32%) species are listed with IUCN Red List status ‘Near Threatened’ or higher including Nepal’s iconic greater one-horned rhinoceros (*Rhinoceros unicornis*), Bengal tiger, Asiatic wild-dog, large Indian civet *(Viverra zibetha)* and fishing cat *(Prionailurus viverrinus)*. Of the mammal species, 24 are within the order Carnivora which could be susceptible to CDV. This area is important in terms of conservation, tourism, and the economy [19, 25, 47–49].

The human population of Chitwan district underwent dramatic growth of 274% between 1920 and 1980. Following a large-scale malaria eradication programme in 1951 and government-induced resettlement of people, the Terai region underwent a period of deforestation, losing 78% of forest cover in 14 years from 1963 with cleared land being mainly used for cultivation [50, 51]. In addition to the national park itself, in 1996 a further 750km^2^ was designated as ‘buffer zone’ (BZ) supporting a population of 260,352 people in 45,616 households [48]. As human populations continue to expand and natural wildlife habitats are reduced and fragmented, contact between human, domestic animal and wildlife populations increases, creating disease interfaces which could promote new opportunities for pathogen transmission affecting all three sectors. [52, 53].

Our research study was conducted over 14 days in November 2019 and focused on domestic dogs in two primary study areas, an urban study area and a buffer zone study area. Bharatpur Metropolitan City (hereafter, “Bharatpur”) was chosen as the urban study area as it supported high densities of free-roaming dogs and was located adjacent to the CNP and buffer zone. With a human population of 280,502 people, Bharatpur is the fifth largest city in Nepal. The main economic sector of the area was originally agriculture, but now combining both rural and urban areas it serves as a commercial centre of Chitwan District and central Nepal [54]. Bharatpur is further divided into administrative wards identified by a numerical system, with the southern and westernmost wards bordering with CNP. Two buffer zone study areas were selected due to their proximity and connectivity to the national park; Sauraha (within Ratnanagar CNP BZ region, human population of 2,699 people) and Jagatpur (within Bharatpur ward-23, in the Kasara Forest region of CNP BZ, human population of 6,878 people) [54]. Serological assays were carried out at the Agriculture and Forestry University (AFU), Rampur Campus, Chitwan, Nepal (Fig 1).

### Ethical approval

Ethical approval (Reference: OS10-19) was granted on 11 September 2019 by the Animal Welfare and Ethical Review Board at the University of Edinburgh. In-country permission was granted from the Agriculture and Forestry University, Faculty of Animal Science, Veterinary Science and Fisheries, Rampur, Chitwan, Nepal.

### Sample collection

A cross-sectional serosurvey was carried out on 100 free-roaming dogs from the study area between 3 and 17 November 2019. Full census data were not available for the free-roaming dog population in all areas of Chitwan, so it was not possible to generate a sample frame from which a randomised survey could be designed. Therefore, the sample population for the study was represented by two sampling groups [55]. The first sample group comprised of opportunistic sampling of a surgical group of 60 free-roaming dogs from Bharatpur and Sauraha, which was carried out alongside a free-roaming dog rabies vaccination and neutering training programme that took place at AFU, Rampur campus. One further female neutered dog was included which had attended the programme for veterinary treatment. As this study utilised opportunistic sampling and did not allow for random field sampling from all study areas, a second field-sampled group of 40 dogs from Jagatpur was also included to reduce sampling selection bias [56, 57], as only entire (un-neutered) dogs were included in the surgical sample group. Additional field sampling was restricted to Jagatpur due to logistical and budgetary limitations.

Dogs from the surgical sample group were sampled whilst under total intravenous general anaesthesia for neutering surgery. The field sampling location, Jagatpur, was visited in early morning when highest numbers of dogs were expected to be encountered [40]. Each dog encountered in the community was caught by hand and muzzled before conscious sample collection. All dog handling and sampling was carried out under veterinary supervision.

The jugular vein was used in the surgical sample group (as peripheral veins were reserved for placement of total intravenous anaesthesia catheters) and the cephalic vein in the field sample group for collection of up to 3ml of blood following venepuncture site preparation.

Collected blood samples were transferred to plain serum vacutainers. All samples were stored in a refrigerator between 2–8°C within four hours of collection, and serum was separated by centrifugation and tested within four days of collection as per the test manufacturer’s guidelines for testing with ***FAST*est**® **CDV** Ab (MEGACOR Diagnostik GmbH, Hoerbranz, Austria) (S1 File). Following neutering surgery each dog was permanently marked by an ear notch, and in the field sample group marked with a non-toxic livestock crayon marker after sampling to avoid accidental re-capture.

### Ancillary data collection

Data were collected for each sampled dog including sex, age, weight in kilograms, body condition score (BCS), ward of residence and clinical signs that could indicate CDV infection (including presence of nasal discharge, ocular discharge, or neurological signs). It is noted that these clinical signs are non-specific and could manifest with other disease presentations.

Sex status was recorded based on visible testes in males and the absence of an ear notch in both males and females. Ear notching following neutering is widely accepted in the area and was 100% accurate for determining entirety prior to sterilisation surgery in both males and females within the surgical sample group.

Age estimate was categorized as juvenile or adult based on the presence of full adult dentition, typically acquired by six months of age. Dogs estimated to be under 16 weeks of age which could have maternally derived CDV antibodies were excluded from the study, with a dental assessment for presence of full adult incisors used to determine age greater than 16 weeks [58, 59].

Bodyweight was measured in kilograms using weighing scales for the surgical sample group and estimated visually, being agreed upon by two team members, for the field sample group. BCS was measured using the WSAVA scoring criteria (BCS 1–9, where 1 is emaciated, 4 or 5 are ideal, and 9 is morbidly obese) [60].

As some dogs from the surgical group were brought directly by community members, individual dog GPS locations were not available. Residence co-ordinates were therefore estimated using ward centroid GPS co-ordinates as obtained from dogdata.uk which logs and maps available census, vaccination and neutering data from contributors working with free-roaming dogs in Nepal [61] (S1 Table). Distance was calculated from each sample ward centroid location to the boundary of CNP (S3 File) (d).

### Laboratory analysis

The ***FAST*est**® **CDV** Ab lateral flow assay has a reported 99.9% sensitivity and 99.8% specificity (S2 File). This test was stable at room temperature during both transportation and storage and can be used without any additional equipment or facilities making it a robust choice for field testing in a basic resource setting. Sample analysis was carried out as per the manufacturer’s guidelines with results interpreted by colour change in relation to a control line as negative, positive medium titre and positive high anti-CDV IgG titre by qualitative colour change with a positive result reported to correspond to a serum neutralisation titre of > 1:16.

### Statistical analysis

Statistical analysis and modelling were conducted using statistical software package R (version 3.6.3, 2018) [62]. Mapping was conducted using QGIS, (version 3.4.2, 2018) [63]. Seroprevalence with 95% confidence intervals (CI) was calculated from a binominal exact test using the ‘epitools’ and ‘epidemr’ packages and plots were created using ggplot2. True prevalence, adjusted for the test sensitivity and specificity, was calculated using the ‘epitools’ package.

Associations between explanatory variables and CDV seropositivity were investigated using univariable logistic regression models with binomial errors. Distemper titre (positive or negative status) was used as the response variable and modelling was performed using the generalised linear model (glm) with logit function in R. Model outputs were summarised using the ‘epiDisplay’ package (S3 File). Significance of association between explanatory variables and CDV seroprevalence was assessed using the likelihood ratio (LR) test and presented as the probability (p) value. The likelihood of positive seroprevalence within variable categories was presented in comparison to the referent category as the odds ratio (OR) with significance assessed by the Wald’s statistic and presented as the probability (p) value. Multivariable logistic regression models were investigated for explanatory variables from univariable outputs with a p-value of < 0.2, and location was included as a random effect. Models were also investigated separately by logistic regression for the surgical sample group and the field sample group. Where standard errors were inflated due to partial or complete separation, Firth regression was used. For all analyses statistical significance was accepted at the 95% confidence level (alpha = 0.05).

Explanatory variables included were sex, age, BCS, presence of clinical signs associated with CDV infection and sampling group. BCS was analysed as low (<4/9) or equal/greater than ideal (≥4/9) to assess adequate nutrition levels and influence on seroprevalence or evidence of clinical disease. Clinical signs were encountered infrequently and so, parameters were grouped for dogs showing one or any combination of signs. Sampling group was analysed as surgical or field sampling groups. Spatial variables considered were sample location in relation to the buffer zone (BZ) and distance from CNP boundary. Ward centroid co-ordinates of sampling locations were calculated using geometry tools in QGIS (S1 Table) and plotted in QGIS to determine sampling location in relation to the BZ. Distance of sampling location to CNP boundary was calculated in QGIS using the ‘measure line’ function within the ‘attribute toolbar’ measuring from ward centroid co-ordinates for each sample location to the closest point of the CNP boundary. Seroprevalence with CI were calculated for each location and associated distance from CNP boundary (S3 File) (d). Variables and association with anti-CDV antibodies were displayed graphically to explore patterns in the data.

## Results

### Demographics of free-roaming dog population

For the 100 dogs sampled, sex was almost evenly split with 52% female and 48% males. Age was heavily skewed with 96% adults to only 4% juvenile. Most dogs (62%) were in good body condition. Clinical signs that may have been attributable to CDV infection were observed in 9% of dogs, with one dog showing both ocular discharge and neurological signs, six dogs showing ocular discharge only, and two showing neurological signs only. Nasal discharge was not detected in any of the dogs sampled. The surgical sampling group represented 60% and the field-group 40% of dogs, with 51% of resident locations outside of the buffer zone in the urban Bharatpur sampling area, and 49% inside the buffer zone. (Table 1).

**Table 1:** Ancillary data collected from the free-roaming domestic dog population surrounding Chitwan National Park and seroprevalence to CDV associated with each of the recorded data variables.

Variable	Number positive	Total	Prevalence %	Lower 95% confidence interval	Upper 95% confidence interval
***Sex***					
*F*	46	52	88.5	76.6	95.6
*M*	34	48	70.8	55.9	83.0
***Age***					
*Adult*	79	96	82.3	73.2	89.3
*Juvenile*	1	4	25.0	0.63	80.6
***BCS***					
*≥4*	48	62	77.4	65.0	87.1
*Low <4*	32	38	84.2	68.7	94.0
***Clinical signs***					
*Absent*	72	91	79.1	69.3	86.9
*Present*	8	9	88.9	51.8	99.7
***Sampling***					
*Surgical*	47	60	78.3	65.8	87.9
*Field*	33	40	82.5	67.2	92.7
***Location***					
*Inside BZ*	41	49	83.7	70.3	92.7
*Urban/ Outside BZ*	39	51	76.5	62.5	87.2
**TOTAL SAMPLE**	80	100	80	70.81	87.33

### Seroprevalence of CDV

Anti-CDV antibodies were detected in 80.0% (CI: 70.8–87.3) of dogs, which included 35.0% (CI: 25.7–45.2) with medium titre and 45.0% (CI: 35.0–55.3) with high titre. Estimated true prevalence (Blaker CL) was 80.0% (CI: 71.1–86.7). Data collected from the study sample groups were analysed to provide baseline demographic information on the free-roaming dog population which was further analysed for CDV seroprevalence (Table 1).

#### Sex demographic

The overall study population comprised of 52% females and 48% males with seroprevalence of 88.5% (CI: 76.6–95.6) and 70.8% (CI: 55.9–83.0) respectively (Table 1). With the exception of the one neutered female dog which was included in the surgical sample group, all other dogs in both the surgical and field sample groups were classed as entire (un-neutered). The surgical sample group comprised of 56.7% females (n = 34) and 43.3% males (n = 26). The field sample group comprised of 45% females (n = 18) and 55% males (n = 22).

#### Age demographic

Within the study population 96% of sampled dogs were adult and 4% juvenile with seroprevalences of 82.3% (CI: 73.2–89.3) and 25.0% (CI: 0.63–80.6) respectively (Table 1). The surgical sample group comprised of 96.7% adults (n = 58) and 3.3% juvenile (n = 2). The field sample group comprised of 95% adults (n = 38) and 5% juvenile (n = 2).

### Variables associated with CDV seroprevalence

For the explanatory variables assessed by univariable logistic regression models for association with positive CDV seroprevalence, only sex and age were associated with positive anti-CDV antibodies indicating past exposure. No statistically significant associations were observed for any other variables (Table 2).

**Table 2:** Association of variables with CDV seropositivity.

Explanatory variable (with positive distemper titre as the response variable)	P value (LR test)	Odds ratio	95% Confidence interval	P value (Wald’s test)
***Sex*** *(Referent category female)*	0.026			
*male*	-	0.32	0.11, 0.91	0.033
***Age*** *(Referent category juvenile)*	0.015			
*adult*	-	13.94	1.37, 142.29	0.026
***BCS*** *(Referent category ≥ ideal BCS)*	0.404			
*low BCS*	-	1.56	0.54, 4.47	0.412
***Clinical signs*** *(Referent category absent)*	0.457			
*Present*	-	2.11	0.25, 17.93	0.494
***Sample method*** *(Referent category surgical group)*	0.608			
*field group*	-	1.30	0.47, 3.62	0.610
***Sample ward distance from CNP (Km)***	0.606			
*continuous variable*	-	0.96	0.82, 1.12	0.601
***Sample location*** *(Referent category urban/ outside BZ)*	0.367			
*inside BZ*	-	1.58	0.58, 4.27	0.370

Further seroprevalence analysis including a breakdown of sex and age categories was calculated for the three main sampling areas of Bharatpur, Jagatpur and Sauraha (S3 File) (c).

To assess impacts of potential confounding between sex and age, and clustering by location, we ran further multivariable models including both variables together with location as random effect. The odds ratio for age and sex were not significantly altered by the inclusion of the random effect for location and showed no major evidence of confounding (S3 File) (f, g).

To explore potential biases from the sampling strategy and check for robustness in the analysis, we estimated the models separately for the non-random surgical sample group and the random field sample group, and then for all data combined. Seroprevalence analysis comparing sex and age in both sample groups were comparable to the total sample. For analysis of association for sex and age with CDV seropositivity, the effect of sex was found to be similar in both the surgical and field sample groups, but the effect of age was larger in the field sample group. Although dilution of the sample size resulted in loss of statistical significance in the p-value, the direction of the effects remained consistent (S3 File) (h, i, j).

### Spatial distribution of CDV seroprevalence

**Fig 2:**
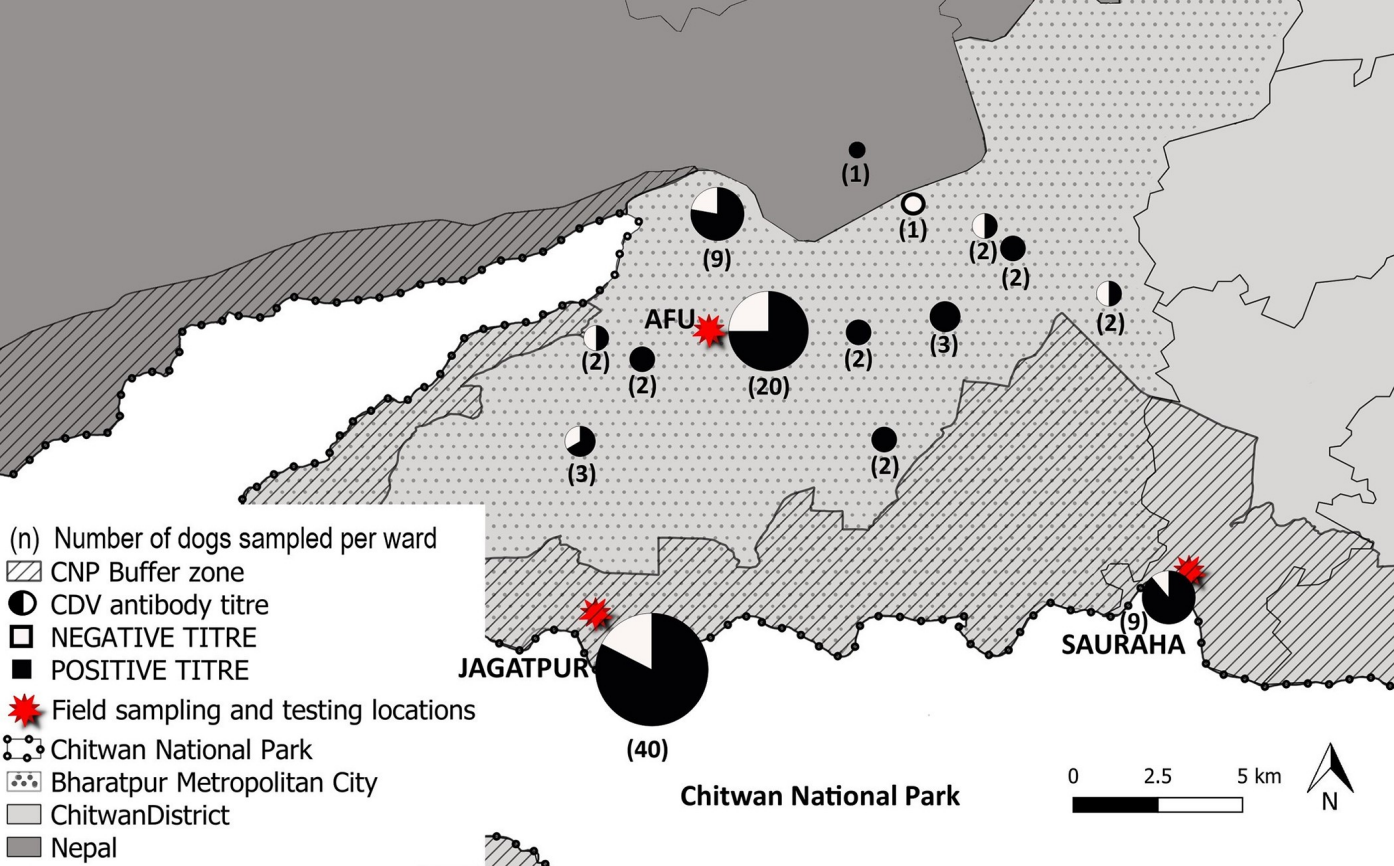
Chitwan District map showing the free-roaming dog canine distemper virus (CDV) seroprevalence results. Field sampling and testing locations are shown in relation to Chitwan National Park (CNP), the buffer zone and Bharatpur Metropolitan City. Seroprevalence results are displayed in pie charts scaled by sample size (black seropositive, white seronegative) at associated ward centroid GPS locations, with the number of dogs sampled from each location displayed in brackets.

Distance from each sample ward centroid location to the boundary of CNP ranged from 0.46–11.39km, with mean 2.97km and SD 2.97 (S3 File) (d). Seroprevalence was mapped by sampling location in relation to CNP and the buffer zone and scaled in pie charts in relation to sample size at each ward location (Fig 2).

## Discussion

The high seroprevalence observed in this study is similar to that of the only previous CDV serosurvey published from Nepal (which found 70% CDV seropositive from the 68 dogs tested) [45], and concurs with other free-roaming dog CDV serosurveys conducted elsewhere in the world [39–43]. High seroprevalence levels could result from a recent outbreak of CDV or could be associated with a widespread endemic disease state. As immunity to CDV is long-lasting, the high seroprevalence demonstrated could limit transmission to low levels, avoiding larger disease outbreaks. However, our findings did confirm CDV exposure in a juvenile dog suggesting recent viral circulation. Further temporal data collection through longitudinal studies, particularly focusing on juvenile dogs, would provide greater understanding of the epidemiology of CDV in the area.

The overall study population had a slightly higher proportion of females (52%) to males (48%), which was contrary to expectation of male sex bias from preference for male dogs for guarding roles and population control in free-roaming dog populations [16, 17, 25]. However, the field sampling group had a higher proportion of males (55%, n = 22), therefore the inclusion of the surgical sampling group may have created selection bias for female dogs. The concurrent neutering programme for the surgical sample group selected only entire dogs, but in the field sampling group, the expectation of the population being largely unneutered [16], was met with 100% of dogs (n = 40) listed as being entire by either the visible presence of testicles in males or the absence of an ear notch in males and females.

Age was heavily skewed towards adult dogs with only 4% being classed as juvenile by the presence of temporary teeth as described by Evans et al. [58]. Litter mortality among free-roaming dogs is typically high with Fiorello at al. [42], and Massei et al. [17], reporting as much as 73% and 60% loss of pups, respectively. Age-related mortality then tends to decrease in dogs over one year old [16, 64]. However, a larger sample size would be required to draw stronger conclusions.

Dogs were mainly in good body condition with ideal or higher BCS seen in 62% and low BCS seen in 38%. This was contrary to expectation for free-roaming dogs but concurred with the findings from Ng et al. [45], from Annapurna, Nepal. Massei et al. [17], surveyed the dog population in Chitwan district during 2013 and determined that the mean age of 3.75 years old was higher than the expectation of other Asian countries. They reported that although most dogs included in the study lacked basic vaccinations and healthcare, 42% of householders interviewed provided food to free-roaming dogs and proposed that population turn-over may not be as high as in other areas. Our BCS findings support this and suggest that positive human relationships and supplementary feeding may be contributing to increased longevity in the vicinity of CNP [15, 17].

Clinical signs that could be attributed to CDV infection were present at low levels (9%). High seroprevalence of 88.8% (CI: 51.7–99.7) was detected in dogs with clinical signs present, although there was no positive association demonstrated by logistic regression. Pathogenicity varies with strain but as many as 50–70% of CDV infections could be sub-clinical [2, 5, 9] and ocular and nasal discharge would only occur during early CDV infection [3, 6]. Positive seroprevalence can also indicate past exposure and represent individuals recovered from infection [8], and the clinical signs we observed could also have been attributable to other non-specific infections.

Representation of the population with the two sampling groups did not significantly affect seroprevalence, with field and surgical sampling group showing 82.5% (CI: 67.2–92.7) and 78.3% (CI: 65.8–87.9) respectively. Therefore, overall seroprevalence was not influenced by sampling bias introduced through the surgical sampling group.

Our study demonstrated, association between sex and CDV prevalence with male dogs having lower seroprevalence than females, and between age and CDV prevalence with adult dogs having higher seroprevalence than juveniles. This was contrary to the study from Annapurna Conservation Area in Nepal [45], which did not demonstrate any associations with seroprevalence. Our study excluded juvenile dogs estimated at under 16 weeks of age, based on dentition, which would exclude disease protection by maternal antibodies in the juvenile group. Higher seroprevalence in adult dogs could be expected with a persistent drive of infection in endemic areas increasing the risk of encountering infection with age, which concurs with other studies [39, 64]. However, our results were based upon small juvenile sample size (n = 4), so a larger sample population would be required to draw stronger conclusions. No significant associations were detected for any other variables examined.

Spatial analysis detected CDV exposure in all but one of the 15 ward sampling locations (Fig 2, S3 File (d)) indicating that CDV is common and widespread in free-roaming dogs surrounding CNP. For the single negative sampling location, exposure may have not been detected due to small sample size (n = 1) at this site. Spatial variables in relation to the urban sampling areas, BZ and distance from CNP boundary did not show a significant association with seroprevalence.

Serosurveys provide baseline data on population demographics and disease prevalence which are useful where data are lacking [40, 65]. However, as seroprevalence can demonstrate past exposure, longer-term data is required to understand epidemic and endemic patterns [8, 28]. Long-term recurring free-roaming dog neutering and rabies vaccination programmes are already active and are ongoing in Nepal. Our study has shown that pathogen surveillance of free-roaming dogs could be integrated into these programmes to provide a valuable source of temporal demographic and pathogen surveillance data.

Our study sample size was small and likely to be biased due to the non-probabilistic study design, despite the inclusion of the field sample group. This means that results may not be representative of the whole population. For example, juvenile dogs were under-represented in the sample. However, seroprevalence from Jagatpur, (the random field-sampled group and largest single sampling area), and the overall study were comparable and sample bias from the opportunistic surgical-sampling group did not appear to affect the overall study outcomes. Single point sampling for CDV prevalence only demonstrates past exposure and not active disease or viral shedding, and longer-term data are required to assess epidemic and endemic disease patterns [8, 28].

Serological tests are unable to differentiate natural wild-type CDV infection from vaccine induced antibody [2, 5, 6]. Enquiries were made to staff from the local non-government veterinary organisations (NGO’s), government veterinary facilities and student veterinary associations in Chitwan to establish potential levels of CDV vaccination within the study area. Based on the records received from all parties, it was established that free-roaming dogs were not routinely vaccinated against CDV, and it was considered unlikely that vaccination has contributed significantly to the high CDV seroprevalence observed in this study.

Virus serum neutralisation (SN) is considered the gold standard serological assay to detect CDV antibodies. Molecular PCR testing can distinguish between vaccine strains and wild type infections [6], and demonstrate gene sequencing associated with active circulating CDV infection [2, 45]. Due to local availability of testing and limitations in logistics and budget, SN and PCR were beyond the scope of this study but would provide a useful focus for future research.

During the neutering and rabies vaccination programme some dogs were brought directly to AFU by community members. As this prevented input of individual dog capture location GPS coordinates from the surgical sample group, data entry method was altered to uniformly record location at ward level. GPS ward centroid coordinates were then used, limiting spatial analysis and mapping of results by ward area rather than by individuals.

There are currently no published reports of CDV in wildlife in Nepal [44], therefore the threat is thus far presumed from cases documented in PAs with similar risks in other countries. A serosurvey of pathogens in eleven free-ranging Bengal tigers in CNP between 2011 and 2017 detected no exposure to CDV, although the small sample size and the importance of continued disease surveillance was acknowledged in the study [46]. Increased tiger mortality has been documented between 2009–2018 in CNP [66]. Although this mortality has not been linked to disease, we have demonstrated high CDV seroprevalence in free-roaming dog populations surrounding CNP, and an awareness of CDV is a critical component of a successful management policy for protected areas containing endangered carnivores [67].

Domestic dogs, being the most abundant susceptible carnivore, are often important reservoirs and sources of infection to threatened populations, and our findings demonstrate widespread high CDV seroprevalence in the free-roaming dog population around CNP. If a disease risk to wildlife was identified, control options of CDV transmission from free-roaming dogs would require consideration. Reducing host density through dog population control has been used to reduce zoonotic disease risk but requires clear understanding of transmission and reservoir systems [68, 69]. Culling to reduce free-roaming dog numbers has been shown to be ineffective as population density recovers rapidly through increased survival or perturbation of the remaining population [69, 70]. Furthermore, use of poisons for population reduction can pose risks to higher endangered trophic levels of predator and scavenger populations [71], and should not be allowed on welfare grounds. For diseases like CDV that evoke prolonged (even life-long) immunity, culling in areas with high seroprevalence, such as we have demonstrated surrounding CNP, can even be counter-productive. Removing herd immunity from past infection with repopulation favouring younger individuals can lead to a naïve, highly susceptible population [72].

Dog density should be managed by changing human attitudes surrounding dog ownership, adequate rubbish disposal, and through fertility control by sterilisation programmes or development of immunocontraceptives [68]. Nepal has numerous NGO’s already working in humane dog population management that could work collaboratively with PA managers. Successful control must balance ecological and cultural needs as poorly managed interventions could result in negative attitudes by local people to further conservation actions [23].

Canine distemper virus is endemic in much of the world. Dog vaccination has seen success since 1960’s leading to control in some countries [10, 14], and rinderpest in artiodactylids was eradicated through vaccination. There are challenges in vaccinating wildlife meaning that most vaccine efforts focus on domestic dog reservoirs. However, although this benefits dogs, difficulties can arise achieving coverage targets and this approach fails to address complex wildlife reservoirs [2]. Belsare & Gompper [73], found that in areas with high CDV seroprevalence, as most adults had already been exposed to the virus, dog vaccination programmes failed to significantly improve the proportion of adult dogs with antibodies. Therefore, in areas such as our study location with high CDV exposure in adult dogs, who would be expected to maintain immunity, the effort, and costs of CDV control programmes in domestic dogs could be better aimed at juvenile animals to reduce pathogen circulation.

Multi-host pathogens like CDV can have complex reservoir systems and wildlife species have also been shown to be capable of maintaining CDV on their own, or as part of a reservoir community, acting as an important source of infection to large carnivores [67]. A ten-year study in Kenya investigated serological exposure patterns to CDV and rabies in domestic dogs, small wild carnivores, and lions [74]. The study proposed that CDV may require larger reservoir populations than rabies, involving either large dog populations or connected cross-species maintenance communities. Phylogenetic studies from dogs and wildlife in the Russian Far East also showed wildlife species to be a more important source of CDV maintenance and infection to Amur Tigers than domestic dogs [67]. In these situations, control focussed on domestic dog vaccination alone would be insufficient to protect endangered wildlife. Chitwan National Park provides habitat for many small mammals including golden jackal *(Canis aureus)*, Bengal foxes *(Vulpes bengalensis)* and mongoose species [48], which can easily move across the park boundary. The common leopard *(Panthera pardus)* is the most common carnivore involved in human-wildlife conflicts in Nepal [75], which often occur in domestic settlements outside of PAs. Further study into wildlife movements, particularly at disease interfaces with domestic species, and cross-species pathogen surveillance including publicly available post-mortem examination findings could provide better understanding of CDV ecology in this area.

Long-term, recurring free-roaming dog population management programmes in the areas surrounding CNP and other PAs provide opportunity to limit dog population size and collect long-term surveillance data to improve understanding of the epidemiology of CDV and other pathogens of free-roaming dogs. If indicated, they are also well placed to provide concurrent ring-vaccination against CDV, particularly to naïve young dogs identified for neutering each year, which in addition to benefiting the dog’s health, could reduce pathogen transmission risk to wildlife in the adjacent PA. Cross-species surveillance allows for assessment of whole ecosystem health and disease control options [8, 76, 77], and understanding of disease reservoir systems should form the foundation of management decisions involving multi-host pathogens [37, 78, 79].

## Supporting information

S1 File FASTest® CDV antibody test instructions for use. (PDF)

S2 File MEGACOR FASTest® CDV Ab internal comparison study. (PDF)

S3 File R raw data. (DOCX)

S4 File Full data set. (CSV)

S1 Table Bharatpur ward centroid GPS coordinates and dog population estimates. (Provided by dogdata.uk). Human ward population 2016 (Provided by bharatpurmun.gov.np).
(DOCX)
