# Supplementary material for: Seroprevalence of canine distemper virus (CDV) in the free-roaming dog (Canis familiaris) population surrounding Chitwan National Park, Nepal
Source: PLoS One. 2023 Feb 27;18(2):e0281542. doi: 10.1371/journal.pone.0281542 (PMC9970093; doi:10.1371/journal.pone.0281542)
Supplement: S1 File — (PDF) [file pone.0281542.s001.pdf]

Version 12/2015

Click Here For More Information About *FASTest*® CDV Ab

[redacted]

ad us. vet.

***In vitro* diagnosticum**

Test-kit for the qualitative detection of antibodies against Canine Distempervirus in whole blood, plasma or serum of the dog

INSTRUCTIONS FOR USE

Supplied Exclusively To The UK Veterinary Market By Vetlab Supplies Ltd
Visit Our Website
www.vetlabsupplies.co.uk
Telephone: 01798 874567
Email us: info@vetlabsupplies.co.uk

[redacted]

1. INFORMATION ON THE TEST-KIT

TEST-KIT COMPONENTS

1 test-kit ***FASTest*® CDV** Ab contains:

- 2 or 10 test cassettes coated with recombinant antigens
- 2 or 10 buffer diluent tubes **A** with 1.0 ml buffer diluent each
- 2 or 10 disposable plastic pipettes (5 µl with mark)
- 2 or 10 disposable plastic pipettes
- 1 instructions for use

STABILITY AND STORAGE

15–25°C Store at 15–25°C

Expiry date – see label

APPLICATION AND ABBREVIATIONS

For veterinary use only

LOT Lot number

In vitro diagnosticum

Do not use test-kit components from different kits, lot numbers or beyond stated expiry date.

Follow instructions for use precisely

T – TEST line, **C** – CONTROL line, **LF** – Lateral flow

LIABILITY

The entire risk due to the performance of this product is assumed by the purchaser. The manufacturer shall not be liable for indirect, special or consequential damages of any kind resulting from the use of this product.

2. INTRODUCTION

Antibodies are basic modules of the humoral immune response. They are passed by passively via the colostrum as so-called maternal antibodies (mAb) onto the yet immunoincompetent newborns or induced actively by natural field infection or vaccination. The antibody titre is varying individually in each animal, depending on multiple factors. The titre can persist over an extended period of time, partially lifelong, in efficient protection concentration (= reliable immunity by protective antibodies) or can fall below the efficient protection concentration (non-reliable immunity) in the course of time.

Depending on the level of individual antibody titre, the veterinarian is able to decide fast and reliable the necessity of vaccination or non-vaccination due to following questions:

Individual vaccination point of the breeding bitch: In problematic breedings, the determination of antibody status of the female makes sense during pregnancy to decide whether a booster vaccination before birth is necessary or to find the optimal primary vaccination time of the puppies.

Individual vaccination point of the puppies: Primary vaccination. There is a critical stage (so-called immunity gap) in puppies, especially in the first 12 weeks. During this stage the concentration of mAb could be high enough to inactivate the vaccinating virus but also too low to protect from field infection. Therefore it is important to find the individual primary vaccination point for each puppy to guarantee an appropriate protection.

For the determination of antibody status of the whole litter, it is possible to determine the antibody status of only one puppy, representative for the other puppies (so-called "fraternal antibody titre"). Here, the balanced colostrum assumption or development of all puppies is absolutely necessary.

Booster vaccination. By determination of the actual antibody status, an individual decision of the necessity of booster vaccination of the puppy or the adult animal can be made.

Being fast, safe and reliable, for pet owner and breeder these important questions can be answered practically by ***FASTest*® CDV** Ab. This enables the veterinarian an appropriate and customized vaccination diagnostics and strategy, adapted to dog and pet owner.

3. INFORMATION ON THE SPECIMEN MATERIAL

Approximately **5 µl (of attached plastic pipette with mark) 15–25°C warm whole blood (WB**, native blood with anticoagulant**), plasma (P) or serum (S)** are needed. **Native blood without anticoagulant should not be used due to potential micro agglutination** (e.g. migration delay on the membrane, unspecific reaction)**!**

Mix the sample material well before use!

Non-cooled (**15–25°C**), WB, P and S should be tested within 4 hours! At **2–8°C**, WB, P and S can be stored up to 4 days. **Serum and/or plasma samples** can be permanently stored **at minimum −20°C.**

Keep in mind that the sample material, as well as all used test-kit components, should have reached room temperature at the time of application.

Endogeneous and exogeneous interfering substances of the sample (e.g. albumin, fibrinogen, lipids, CRP, heterophilic antibodies, especially type IgA, as well as viscosity, pH-value and excess EDTA) **as well as native blood can cause interferences** (matrix effects) **that can influence the target measurement. These can lead to an impaired LF and/or unspecific reactions on T and C.**

4. SPECIMEN COLLECTION AND PREPARATION

a. Draw sample **up to the mark (≙ 5 µl sample volume)** using the disposable 5 µl plastic pipette (fig.1).
b. Open the cap of the buffer diluent tube **A** and mix the 5 µl of the sample by repeatedly press and release of the pipette into the buffer diluent (fig.2). Discard the pipette.
c. Close the buffer diluent tube **A** well. Mix the sample-buffer mixture (SBM) homogeneously by careful swinging.

5. TEST PROCEDURE

1. Remove the test cassette from its foil pouch shortly before use. Place it on a flat surface.
2. Open the buffer diluent tube **A** containing the SBM. Place **4 drops (ca. 160–200 µl) of the SBM** slowly into the sample window S of the test cassette using the disposable plastic pipette (without mark; hold pipette vertically, fig.3).
3. Add 1 additional drop of SBM into the sample window S if there is no beginning pink-purple LF visible within 1 minute after adding the SBM.

5 µl →

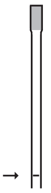

fig.1

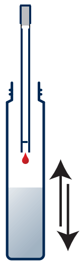

5 µl whole blood, plasma or serum

fig.2

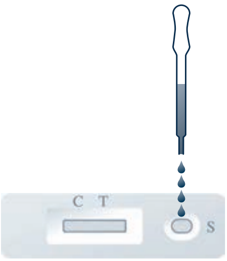

fig.3

6. READING OF THE TEST RESULT

10 min

Read the test result **10 minutes** after addition of the SBM into the sample window S.

POSITIVE TEST RESULT

(prolonged vaccination interval possible)

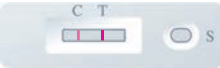

HIGH TITRE
(≈ SN* titre > 1:64)
Colour intensity **T** > **C**

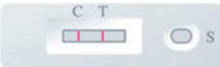

MEDIUM TITRE
(≈ SN titre ≥ 1:16, < 1:32)
Colour intensity **T** ≙ **C**

NEGATIVE TEST RESULT

(Distempervirus vaccination recommended)

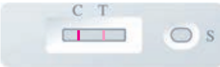

LOW or NO TITRE
(≈ SN titre < 1:16)
Colour intensity **T** < **C** or **T** not visible

INVALID TEST RESULT

Only a weak to strong and well-defined pink-purple TEST line or no line at all appears. The test should be repeated using a new test cassette.

* SN titre from serum neutralisation test

7. PRECAUTIONS FOR USERS

- Label sample material and associated test cassette to ensure a precise assignment.
- Use a new buffer diluent tube and new pipettes for each sample.
- The ***FASTest*® CDV** Ab is not suitable for the detection of Distempervirus IgG antibodies in cats.
- **ATTENTION**: Partially filled and/or insufficient mixed EDTA, Citrate or Heparin tubes could create invisible microclots resulting in lateral flow delay and/or unspecific reactions (e.g. greyish shadow like lines).
- The buffer diluent contains low concentrations of toxic sodium azide as a preservative, therefore avoid skin contact and/or ingestion.
- The sample material must be seen as potentially infectious and disposed of accordingly, together with the used test-kit components.

8. TEST PRINCIPLE

The ***FASTest*® CDV** Ab is based on an immunochromatographic "sandwich principle".

The anti-CDV antibodies of the sample first react with the recombinant CDV antigens of the sample pad, second with the mobile monoclonal gold labeled antibodies of the conjugate pad. During the following "lateral flow" (**LF**) along the nitrocellulose membrane, these antigen-antibody complexes are captured by fixed polyclonal antibodies forming a pink-purple TEST line **T**. The colour intensity of T can vary depending on the anti-CDV antibody concentration of the sample.

A correct test procedure will be indicated by a second, clearly outlined pink-purple CONTROL line (**C**).

Evaluation of ***FASTest*® CDV** Ab is done by comparison of the colour intensities of T with C.

The threshold titre (sustainable immunity or not) of ***FASTest*® CDV** Ab (1:16) is adjusted by Golden Standard Test (serum neutralisation test).

9. INFORMATION FOR THE INTERPRETATION

- The interpretation of the test result should always be based on anamnestic and clinical data as well as the therapy and prophylaxis possibilities.
- Any non-described colour or contour variation of T and C (e.g. greyish, shadow-like lines) has to be considered as unspecific reactions and therefore as negative test result.
- Due to anticoagulated whole blood and/or red hemoglobin background of the test membrane caused by hemolytic blood samples, the visibility of T, especially in case of weak positive samples, could be from worse to not visible.
- Any coloured lines appearing after 20 minutes do not have any diagnostic value.
- The ***FASTest*® CDV** Ab only detects the presence or absence of anti-CDV IgG antibodies in the specimen and should not be used as the sole criterion for the diagnosis of the CDV immune status in dogs.

Protective Distemper antibody titre ≥ 1:16

- high to medium titre: indicative of a very good to good CDV immune status ➔ prolonged vaccination interval possible, repetition of ***FASTest*® CDV** Ab every 6 months recommended
- low to no titre: indicative of a bad to very bad CDV immune status ➔ **immediate vaccination recommended!**

6912 Hörbranz – AUSTRIA
www.megacor.com
