# Supplementary material for: Seroprevalence of canine distemper virus (CDV) in the free-roaming dog (Canis familiaris) population surrounding Chitwan National Park, Nepal
Source: PLoS One. 2023 Feb 27;18(2):e0281542. doi: 10.1371/journal.pone.0281542 (PMC9970093; doi:10.1371/journal.pone.0281542)
Supplement: S2 File — (PDF) [file pone.0281542.s002.pdf]

[redacted]
Lochauer Str. 2
A–6912 Hörbranz–AUSTRIA
Tel.: +43 5573 85400
Fax: +43 5573 85400-4
www.megacor.com

Diagnostik MEGACOR

Internal comparison study **Date: September 2013**

***FAST*est® CDV AB** versus **serum neutralisation**

	**Serum neutralisation positive**	**Serum neutralisation negative**	
***FAST*est® CDV AB** Test positive	**260** A = TP	**0** B = FP	**260** A+B
***FAST*est® CDV AB** Test negative	**0** C = FN	**26** D = TN	**26** C+D
	**260** A+C	26 B+D	**286** A+B+C+D

SUMMARY:

In total 286 sera (260 positive and 26 negative sera tested with serum neutralisation) were compared.

*FAST*est® CDV AB:

Sensitivity (A/(A+C): proportion of „true positive“ (TP) of all sick animals **100%**

Specificity (D/(B+D): proportion of „true negative“ (TN) of all not sick animals **100%**

Positive Predictive Value: A/(A+B): proportion of TP of all test-positive animals **100%**

Negative Predictive Value: D/(C+D): proportion of TN of all test-negative animals **100%**
