# Supplementary material for: Seroprevalence of canine distemper virus (CDV) in the free-roaming dog (Canis familiaris) population surrounding Chitwan National Park, Nepal
Source: PLoS One. 2023 Feb 27;18(2):e0281542. doi: 10.1371/journal.pone.0281542 (PMC9970093; doi:10.1371/journal.pone.0281542)
Supplement: S3 File — (DOCX) [file pone.0281542.s003.docx]

**FULL STATISTICAL ANALYSIS AND RAW DATA OUTPUTS FROM R**

1. Overall seroprevalence with 95% CI

| **Variable** | **No. +ve** | **No. Total** | **Prevalence** | **Lower CI** | **Upper CI** |
| --- | --- | --- | --- | --- | --- |
| Total sample | 80 | 100 | 80 | 70.81573 | 87.33444 |
| Negative titre | 20 | 100 | 20 | 12.66556 | 29.18427 |
| Medium titre | 35 | 100 | 35 | 25.72938 | 45.18494 |
| High titre | 45 | 100 | 45 | 35.03202 | 55.27198 |

1. Ancillary demographic data and seroprevalence with 95% CI

| **Variable** | **No. +ve** | **No. total** | **Prevalence** | **Lower CI** | **Upper CI** |
| --- | --- | --- | --- | --- | --- |
| **Sex** |  |  |  |  |  |
| M | 34 | 48 | 70.83333 | 55.93652 | 83.04692 |
| F | 46 | 52 | 88.46154 | 76.55917 | 95.64588 |
| **Age** |  |  |  |  |  |
| Adult | 79 | 96 | 82.29167 | 73.16814 | 89.33230 |
| Juvenile | 1 | 4 | 25.00000 | 0.630946 | 80.58796 |
| **BCS** |  |  |  |  |  |
| Low <4 | 32 | 38 | 84.21053 | 68.74664 | 93.97703 |
| >=4 | 48 | 62 | 77.41935 | 65.02631 | 87.06961 |
| **Clinical signs** |  |  |  |  |  |
| Present | 8 | 9 | 88.88889 | 51.75035 | 99.71909 |
| Absent | 72 | 91 | 79.12088 | 69.33080 | 86.93622 |
| **Sampling** |  |  |  |  |  |
| Random | 33 | 40 | 82.50000 | 67.22099 | 92.66173 |
| Convenience | 47 | 60 | 78.33333 | 65.80438 | 87.92840 |
| **Location** |  |  |  |  |  |
| Inside BZ | 41 | 49 | 83.67347 | 70.34286 | 92.67772 |
| Outside BZ | 39 | 51 | 76.47059 | 62.50695 | 87.20919 |

1. Seroprevalence to CDV split spatially between sampling areas with further breakdown of sex and age categories.

|  | **Number positive** | **Total** | **Prevalence %** | **Lower 95% confidence interval** | **Upper 95% confidence interval** |
| --- | --- | --- | --- | --- | --- |
| **BHARATPUR SAMPLE (all other wards)** | 39 | 51 | 76.0 | 62.5 | 87.2 |
| **Variable** |  |  |  |  |  |
| ***Sex*** |  |  |  |  |  |
| F | 26 | 31 | 83.9 | 66.3 | 94.5 |
| M | 13 | 20 | 65.0 | 40.8 | 84.6 |
| **Age** |  |  |  |  |  |
| Adult | 39 | 50 | 78.0 | 64.0 | 88.5 |
| Juvenile | 0 | 1 | 0.0 | 0.0 | 97.5 |
|  |  |  |  |  |  |
| **JAGATPUR SAMPLE (ward-23)** | 33 | 40 | 83.0 | 67.2 | 92.7 |
| **Variable** |  |  |  |  |  |
| **Sex** |  |  |  |  |  |
| F | 17 | 18 | 94.4 | 72.7 | 99.9 |
| M | 16 | 22 | 72.7 | 49.8 | 89.3 |
| **Age** |  |  |  |  |  |
| Adult | 33 | 38 | 86.8 | 71.9 | 95.6 |
| Juvenile | 0 | 2 | 0.0 | 0.0 | 84.2 |
|  |  |  |  |  |  |
| **SAURAHA SAMPLE** | 8 | 9 | 89.0 | 51.8 | 99.7 |
| **Variable** |  |  |  |  |  |
| **Sex** |  |  |  |  |  |
| F | 3 | 3 | 100 | 29.2 | 100 |
| M | 5 | 6 | 83.3 | 35.9 | 99.6 |
| **Age** |  |  |  |  |  |
| Adult | 7 | 8 | 87.5 | 47.3 | 99.7 |
| Juvenile | 1 | 1 | 100.0 | 2.50 | 100 |

1. Seroprevalence with 95% CI based on distance from CNP

| **Location – ward number** | **Km from CNP** | **Number positive** | **Total** | **Prevalence** | **Lower confidence interval** | **Upper confidence interval** |
| --- | --- | --- | --- | --- | --- | --- |
| Sauraha | 0.46 | 8 | 9 | 88.89 | 51.75 | 99.72 |
| Bharatpur-17 | 0.75 | 1 | 2 | 50.00 | 1.25 | 98.74 |
| Bharatpur-23 | 0.83 | 33 | 40 | 82.50 | 67.22 | 92.66 |
| Bharatpur-16 | 1.53 | 7 | 9 | 77.78 | 39.99 | 97.18 |
| Bharatpur-19 | 2.71 | 2 | 2 | 100.00 | 15.81 | 100.00 |
| Bharatpur-20 | 4.57 | 2 | 3 | 66.67 | 9.43 | 99.16 |
| Bharatpur-15 | 4.78 | 15 | 20 | 75.00 | 50.90 | 91.34 |
| Bharatpur-6 | 5.38 | 2 | 2 | 100.00 | 15.81 | 100.00 |
| Gaindakot | 6.53 | 1 | 1 | 100.00 | 15.81 | 100.00 |
| Bharatpur-5 | 7.41 | 2 | 2 | 100.00 | 15.81 | 100.00 |
| Bharatpur-3 | 7.80 | 0 | 1 | 0 | 0.00 | 97.50 |
| Bharatpur-12 | 9.15 | 1 | 2 | 50.00 | 1.25 | 98.74 |
| Bharatpur-7 | 9.44 | 3 | 3 | 100.00 | 15.81 | 100.00 |
| Bharatpur-10 | 10.03 | 1 | 2 | 50.00 | 1.25 | 98.74 |
| Bharatpur-HART office | 11.39 | 2 | 2 | 100.00 | 15.81 | 100.00 |

1. Univariable logistic regression with positive distemper titre as the response variable and X as the explanatory variable using epiDisplay model in R studio. Full raw data output.

|  | **Estimate std.** | **OR** | **SE** | **95%CI** | **z** | **p** | **p(Wald’s test)** | **p (LR test)** |
| --- | --- | --- | --- | --- | --- | --- | --- | --- |
| (Intercept) | 2.0369 | 7.67 | 3.33 |  | 4.69 | <.001 |  |  |
| **SEX sex2M** | -1.1496 | 0.32 | 0.17 | (0.11, 0.91) | -2.14 | 0.033 | 0.033 | 0.026 |
| (Intercept) | 1.099 | 0.33 | 0.38 |  | -0.95 | 0.341 |  |  |
| **AGE Age.adult** | 2.635 | 13.94 | 16.52 | (1.37-142.29) | 2.22 | 0.026 | 0.026 | 0.015 |
| (Intercept) | 1.2321 | 3.43 | 1.04 |  | 4.06 | <.001 |  |  |
| **BODY CONDITION Bcs.low** | 0.4418 | 1.56 | 0.84 | (0.54-4.47) | 0.82 | 0.412 | 0.412 | 0.404 |
| (Intercept) | 1.3322 | 3.79 | 0.98 |  | 5.17 | <.001 |  |  |
| **CLINICAL SIGNS Clinical.signs** | 0.7472 | 2.11 | 2.30 | (0.25-17.93) | 0.68 | 0.494 | 0.494 | 0.457 |
| (Intercept) | 1.1787 | 3.25 | 1.07 |  | 3.57 | <.001 |  |  |
| **LOCATION Buffer.zone** | 0.4555 | 1.58 | 0.80 | (0.58-4.27) | 0.90 | 0.370 | 0.37 | 0.367 |
| (Intercept) | 1.2852 | 3.62 | 1.13 |  | 4.10 | <.001 |  |  |
| **SAMPLE UNIT Sampling.random** | 0.2654 | 1.30 | 0.68 | (0.47-3.62) | 0.51 | 0.610 | 0.61 | 0.608 |
| (Intercept) | 1.51723 | 4.56 | 1.64 |  | 4.21 | <.001 |  |  |
| **DISTANCE CNP Cnp.km** | -0.04253 | 0.96 | 0.08 | (0.82-1.12) | -0.52 | 0.601 | 0.601 | 0.606 |

1. Multivariable logistic regression with positive distemper titre as the response variable and X as the explanatory variable. Run for explanatory variables from the univariable model with a p-value of < 0.2.

|  | **Estimate std.** | **OR** | **SE** | **95%CI** | **z** | **p** |
| --- | --- | --- | --- | --- | --- | --- |
| (Intercept) | -0.1603 | 0.85 | 1.09 | (0.04-8.87) | -0.13 | 0.900 |
| **SEX sex2M** | -0.9383 | 0.39 | 0.22 | (0.12-1.13) | -1.69 | 0.092 |
| **AGE age.adult** | 2.1972 | 9.00 | 10.85 | (1.03-192.75) | 1.82 | 0.068 |

1. Random effects models for location.

Generalised linear model with positive distemper titre as the response variable and sex as the explanatory variable and with random effects for location.

| **FIXED EFFECTS** | | | | | | | | |
| --- | --- | --- | --- | --- | --- | --- | --- | --- |
| **Parameter** | **Odds Ratio** | | **SE** | **95% CI** | | **z** | | **p** |
| **(Intercept)** | 7.67 | | 3.33 | [3.27, 17.95] | | 4.69 | | < .001 |
| **SEX sex2 (m)** | 0.32 | | 0.17 | [0.11, 0.91] | | -2.14 | | 0.033 |
| **RANDOM EFFECTS** | | | | | | | | |
| **Parameter** | | **Coefficient** | | | **SE** | | **95% CI** | |
| **SD (Intercept: location)** | | 3.02e-08 | | | 0.34 | | [0.00, Inf] | |
| **SD (Residual)** | | 1.00 | | |  | |  | |
| **Wald z- distribution approximation** | | | | | | | | |
| **Parameter** | **Odds Ratio** | | **SE** | **95% CI** | | **z** | | **p** |
| **(Intercept)** | 7.67 | | 3.33 | [3.54, 20.03] | | 4.69 | | < .001 |
| **SEX sex2 (m)** | 0.32 | | 0.17 | [0.10, 0.88] | | -2.14 | | 0.033 |

Generalised linear model with positive distemper titre as the response variable and age as the explanatory variable and with random effects for location.

| **FIXED EFFECTS** | | | | | | | | |
| --- | --- | --- | --- | --- | --- | --- | --- | --- |
| **Parameter** | **Odds Ratio** | | **SE** | **95% CI** | | **z** | | **p** |
| **(Intercept)** | 0.33 | | 0.38 | [0.03, 3.20] | | -0.95 | | 0.341 |
| **AGE Age.adult** | 13.94 | | 16.52 | [1.37, 142.29] | | 2.22 | | 0.026 |
| **RANDOM EFFECTS** | | | | | | | | |
| **Parameter** | | **Coefficient** | | | **SE** | | **95% CI** | |
| **SD (Intercept: location)** | | 0.00 | | | 0.35 | | 0.00 | |
| **SD (Residual)** | | 1.00 | | |  | |  | |
| **Wald z- distribution approximation** | | | | | | | | |
| **Parameter** | **Odds Ratio** | | **SE** | **95% CI** | | **z** | | **p** |
| **(Intercept)** | 0.33 | | 0.38 | [0.02, 2.60] | | -0.95 | | 0.341 |
| **AGE Age.adult** | 13.94 | | 16.52 | [1.67, 291.44] | | 2.22 | | 0.026 |

Generalised linear model with positive distemper titre as the response variable and sex and age as the explanatory variables and with random effects for location.

| **FIXED EFFECTS** | | | | | | | | |
| --- | --- | --- | --- | --- | --- | --- | --- | --- |
| **Parameter** | **Odds Ratio** | | **SE** | **95% CI** | | **z** | | **p** |
| **(Intercept)** | 0.85 | | 1.09 | [0.07, 10.51] | | -0.13 | | 0.900 |
| **SEX sex2 (m)** | 0.39 | | 0.22 | [0.13, 1.16] | | -1.69 | | 0.092 |
| **AGE Age.adult** | 9.00 | | 10.85 | [0.85, 95.68] | | 1.82 | | 0.068 |
| **RANDOM EFFECTS** | | | | | | | | |
| **Parameter** | | **Coefficient** | | | **SE** | | **95% CI** | |
| **SD (Intercept: location)** | | 0.00 | | | 0.39 | | 0.00 | |
| **SD (Residual)** | | 1.00 | | |  | |  | |
| **Wald z- distribution approximation** | | | | | | | | |
| **Parameter** | **Odds Ratio** | | **SE** | **95% CI** | | **z** | | **p** |
| **(Intercept)** | 0.85 | | 1.09 | [0.04, 8.87] | | -0.13 | | 0.900 |
| **SEX sex2 (m)** | 0.39 | | 0.22 | [0.12, 1.13] | | -1.69 | | 0.092 |
| **AGE Age.adult** | 9.00 | | 10.85 | [1.03, 192.75] | | 1.82 | | 0.068 |

1. Stratified model for sampling strategy comparing random and non-random using Firth regression

| **FIXED EFFECTS** | | | | | | | | |
| --- | --- | --- | --- | --- | --- | --- | --- | --- |
| **Parameter** | **Odds Ratio** | | **SE** | **95% CI** | | **z** | | **p** |
| **(Intercept)** | 2.39 | | 3.73 | [0.11, 50.89] | | 0.56 | | 0.577 |
| **SEX sex2 (m)** | 0.42 | | 0.28 | [0.11, 1.53] | | -1.32 | | 0.187 |
| **AGE Age.adult** | 2.43 | | 3.60 | [0.13, 44.50] | | 0.60 | | 0.550 |
| **RANDOM EFFECTS** | | | | | | | | |
| **Parameter** | | **Coefficient** | | | **SE** | | **CI low** | |
| **SD (Intercept: location)** | | 0.00 | | | 0.45 | | 0.00 | |
| **SD (Residual)** | | 1.00 | | |  | |  | |
| **FIRTH REGRESSION FIXED EFFECTS** | | | | | | | | |
| **Parameter** | **Odds Ratio** | | **SE** | **95% CI** | | **Chi2 (1)** | | **p** |
| **(Intercept)** | 0.64 | | 1.17 | [0.00, 19.02] | | 0.06 | | 0.803 |
| **SEX sex2 (m)** | 0.31 | | 0.32 | [0.03, 1.94] | | 1.50 | | 0.221 |
| **AGE Age.adult** | 18.33 | | 30.03 | [1.21, 2717.52] | | 4.45 | | 0.035 |

1. Seroprevalence to CDV split by sampling groups with further breakdown of sex and age categories

| **Variable** | **No. +ve** | **No. total** | **Prevalence** | **Lower CI 95%** | **Upper CI 95%** |
| --- | --- | --- | --- | --- | --- |
| **TOTAL SAMPLE** | | | | | |
| **Total** | 80 | 100 | 80 | 70.8 | 87.3 |
| **Sex** |  |  |  |  |  |
| M | 34 | 48 | 70.8 | 55.9 | 83.0 |
| F | 46 | 52 | 88.5 | 76.6 | 95.6 |
| **Age** |  |  |  |  |  |
| Adult | 79 | 96 | 82.3 | 73.2 | 89.3 |
| Juvenile | 1 | 4 | 25.0 | 0.63 | 80.6 |
| **SURGICAL SAMPLE GROUP** | | | | | |
| **Total** | 47 | 60 | 78.3 | 65.8 | 87.9 |
| **Sex** |  |  |  |  |  |
| M | 18 | 26 | 69.2 | 48.2 | 85.7 |
| F | 29 | 34 | 85.3 | 68.9 | 95.0 |
| **Age** |  |  |  |  |  |
| Adult | 46 | 58 | 79.3 | 66.7 | 88.9 |
| Juvenile | 1 | 2 | 50.0 | 1.3 | 98.7 |
| **FIELD SAMPLE GROUP** | | | | | |
| **Total** | 33 | 40 | 82.5 | 67.2 | 92.7 |
| **Sex** |  |  |  |  |  |
| M | 16 | 22 | 72.7 | 49.8 | 89.3 |
| F | 17 | 18 | 94.4 | 72.7 | 99.9 |
| **Age** |  |  |  |  |  |
| Adult | 33 | 38 | 86.8 | 71.9 | 95.6 |
| Juvenile | 0 | 2 | 0.0 | 0.0 | 84.2 |

1. Univariable logistic regression and fishers exact test split by sampling groups

|  | **Univariable logistic regression** | | | | **Fisher’s exact test** | | |
| --- | --- | --- | --- | --- | --- | --- | --- |
| **Explanatory variable** (with positive distemper titre as the response variable) | **P value (LR test)** | **Odds Ratio** | **95% Confidence interval** | **P value (Wald’s test)** | **Odds Ratio** | **95% Confidence interval** | **P value (Fishers exact)** |
| **TOTAL SAMPLE** | | | | | | | |
| ***Sex*** *(Referent category female)* | 0.026 |  |  |  |  |  | 0.04 |
| *male* | - | 0.32 | 0.11, 0.91 | 0.033 | 0.32 | 0.09,0.99 |  |
| ***Age*** *(Referent category juvenile)* | 0.015 |  |  |  |  |  | 0.024 |
| *adult* | - | 13.94 | 1.37, 142.29 | 0.026 | 13.4 | 1.01,737.1 |  |
| **SURGICAL SAMPLE GROUP** | | | | | | | |
| ***Sex*** *(Referent category female)* | 0.136 |  |  |  |  |  | 0.21 |
| *male* | - | 0.39 | (0.11,1.37) | 0.136 | 0.39 | 0.087,1.617 |  |
| ***Age*** *(Referent category juvenile)* | 0.369 |  |  |  |  |  | 0.39 |
| *adult* | - | 3.83 | (0.22,65.85) | 0.369 | 3.72 | 0.045,306.436 |  |
| **FIELD SAMPLE GROUP** | | | | | | | |
| ***Sex*** *(Referent category female)* | 0.058 |  |  |  |  |  | 0.105 |
| *male* | - | 0.16 | (0.02,1.45 | 0.058 | 0.16 | 0.003,1.59 |  |
| ***Age*** *(Referent category juvenile)* | 0.006 |  |  |  |  |  | 0.02692 |
| *adult* | - | 280795761.49 | (0,Inf) | 0.006 | Inf | 0.956, Inf |  |
