# Supplementary material for: Seroprevalence of canine distemper virus (CDV) in the free-roaming dog (Canis familiaris) population surrounding Chitwan National Park, Nepal
Source: PLoS One. 2023 Feb 27;18(2):e0281542. doi: 10.1371/journal.pone.0281542 (PMC9970093; doi:10.1371/journal.pone.0281542)
Supplement: S1 Table — (Provided by dogdata.uk). Human ward population 2016 (Provided by bharatpurmun.gov.np). (DOCX) [file pone.0281542.s005.docx]

**BHARATPUR WARD CENTROIDS AND AVAILABLE DOG AND HUMAN POPULATION ESTIMATES.**

| **DISTRICT** | **MUNICIPALITY** | **WARD** | **LATITUDE** | **LONGITUDE** | **AVAILABLE DOG POPULATION ESTIMATE 2018** | **HUMAN POPULATION 2016** |
| --- | --- | --- | --- | --- | --- | --- |
| Chitwan | Bharatpur Metro | 01 | 27.72654 | 84.45948 | 98 | 10566 |
| Chitwan | Bharatpur Metro | 02 | 27.69960 | 84.43494 | 198 | 16218 |
| Chitwan | Bharatpur Metro | 03 | 27.68961 | 84.40934 | 42 | 14461 |
| Chitwan | Bharatpur Metro | 04 | 27.67386 | 84.39870 | 118 | 14482 |
| Chitwan | Bharatpur Metro | 05 | 27.65027 | 84.39180 | 86 | 7856 |
| Chitwan | Bharatpur Metro | 06 | 27.61817 | 84.39954 | 81 | 10824 |
| Chitwan | Bharatpur Metro | 07 | 27.65402 | 84.41690 | 153 | 10505 |
| Chitwan | Bharatpur Metro | 08 | 27.60526 | 84.43931 | 152 | 6845 |
| Chitwan | Bharatpur Metro | 09 | 27.66115 | 84.44100 | 102 | 9116 |
| Chitwan | Bharatpur Metro | 10 | 27.68200 | 84.42957 | 136 | 21844 |
| Chitwan | Bharatpur Metro | 11 | 27.68882 | 84.45936 | 376 | 21004 |
| Chitwan | Bharatpur Metro | 12 | 27.66177 | 84.46675 | 113 | 10939 |
| Chitwan | Bharatpur Metro | 13 | 27.58864 | 84.38225 | 96 | 5973 |
| Chitwan | Bharatpur Metro | 14 | 27.60862 | 84.36292 | 125 | 8521 |
| Chitwan | Bharatpur Metro | 15 | 27.64242 | 84.35677 | - | 11773 |
| Chitwan | Bharatpur Metro | 16 | 27.68133 | 84.34540 | - | 13694 |
| Chitwan | Bharatpur Metro | 17 | 27.65607 | 84.31339 | - | 6710 |
| Chitwan | Bharatpur Metro | 18 | 27.63585 | 84.29178 | - | 7018 |
| Chitwan | Bharatpur Metro | 19 | 27.64221 | 84.32709 | - | 6175 |
| Chitwan | Bharatpur Metro | 20 | 27.61676 | 84.30783 | - | 6676 |
| Chitwan | Bharatpur Metro | 21 | 27.59930 | 84.32705 | - | 6506 |
| Chitwan | Bharatpur Metro | 22 | 27.57872 | 84.35103 | - | 5269 |
| Chitwan | Bharatpur Metro | 23 | 27.57008 | 84.31690 | - | 6878 |
| Chitwan | Bharatpur Metro | 24 | 27.57327 | 84.28224 | - | 4317 |
| Chitwan | Bharatpur Metro | 25 | 27.58633 | 84.27420 | - | 8057 |
| Chitwan | Bharatpur Metro | 26 | 27.61673 | 84.25768 | - | 8337 |
| Chitwan | Bharatpur Metro | 27 | 27.58579 | 84.23105 | - | 7780 |
| Chitwan | Bharatpur Metro | 28 | 27.57629 | 84.18956 | - | 6369 |
| Chitwan | Bharatpur Metro | 29 | 27.77958 | 84.48314 | - | 5815 |
